# Outcomes of Deep Sclerectomy following Failed XEN Gel Stent Implantation in Open-Angle Glaucoma: A Prospective Study

**DOI:** 10.3390/jcm11164784

**Published:** 2022-08-16

**Authors:** Giorgio Enrico Bravetti, Kevin Gillmann, Harsha L. Rao, André Mermoud, Kaweh Mansouri

**Affiliations:** 1 Glaucoma Research Center, Montchoisi Clinic, Swiss Visio, 1006 Lausanne, Switzerland; 2 Narayana Nethralaya, 63, Bannerghatta Road, Hulimavu, Bangalore 560099, India; 3 Department of Ophthalmology, University of Denver, Denver, CO 80208, USA

**Keywords:** glaucoma, open-angle glaucoma, minimally invasive glaucoma surgery, MIGS, XEN gel stent, non-penetrating glaucoma surgery, deep sclerectomy, safety, secondary procedure

## Abstract

Background: The purpose of this study is to evaluate the outcome of deep sclerectomy (DS) as a secondary procedure following failed ab-interno XEN gel stent implantation in patients with open-angle glaucoma. Methods: Prospective, single-center, non-randomized, interventional study. Consecutive eyes that underwent mitomycin C (MMC) augmented XEN gel stent surgery, with uncontrolled intraocular pressure (IOP) or signs of disease progression, were included to undergo MMC-augmented DS. Primary efficacy outcome was surgical success, defined as complete when the unmedicated IOP was 12 mmHg or less, or 15 mmHg or less and 20% lower than at the timing of XEN failure and defined as qualified when the IOP fulfilled the same conditions with fewer medications than before deep sclerectomy. Secondary measures were mean reduction in IOP and in the number of medications, and the rates of complications. Results: Seventeen eyes were enrolled with a mean age of 72.1 ± 8.2 years (66.7% women). The mean follow-up was 20.1 ± 4.9 months, with more than 12-month data available from 15 eyes. Following DS, IOP decreased significantly from 22.6 ± 5.3 mmHg to 12.3 ± 5.5 (45.6%; *p* < 0.001). Antiglaucoma medications dropped from 1.1 ± 0.9 to 0.3 ± 0.7. Complete success was obtained in 40% of eyes using the threshold of 12 mmHg or less and a 20% decrease of IOP, and in 60% using the 15 mmHg or less threshold. Adverse events were observed in 20% of eyes (bleb leakage (13.3%); hypotony (6.7%)). No cases of choroidal detachment or hypotony maculopathy were reported. Conclusions: Failed XEN gel stent implantation does not seem to negatively affect the safety and efficacy of subsequent deep sclerectomy surgery.

## 1. Introduction

Glaucoma management is currently based on lowering intraocular pressure (IOP) in order to prevent the progressive loss of retinal ganglion cells [1]. In recent years, the development of alternative approaches to traditional filtering surgery has caused a shift in glaucoma management. Minimally invasive glaucoma surgery (MIGS) techniques provide clinicians with a safe, effective, and minimally-invasive surgical alternative, encouraging an early transition from topical therapies to surgery, while delaying, or avoiding, more invasive procedures, such as filtering techniques [2]. The popularity of MIGS is based on the assumption that these procedures have little or no effect on the outcome of subsequent glaucoma surgery. Scarce data, however, are available to support this assumption.

The XEN gel stent (Allergan, Dublin, CA, USA) is one of these surgical options that targets the subconjunctival outflow pathway through an ab-interno placement [3]. It has demonstrated safety and efficacy in lowering IOP in a wide array of situations: as a standalone first-line procedure [4], in eyes with failed prior surgery [5], in combination with cataract surgery [6,7,8], and in eyes with primary open-angle glaucoma (POAG) and pseudo-exfoliative glaucoma (PEXG) [9,10]. In view of these findings, the XEN gel stent is being increasingly used as a surgical approach in early-to-moderate glaucoma. Nevertheless, as a bleb-creating procedure, a failure of the procedure may impair the efficacy of subsequent filtering surgeries that rely on conjunctival integrity. Despite a recent study demonstrating that mitomycin C (MMC)-augmented trabeculectomy following failed XEN gel stent surgery is technically feasible, and a case report wherein XEN-augmented Baerveldt surgery was used to rescue a failed XEN, data on surgeries following failed XEN gel stents remain scarce [11,12].

The aim of this study was to assess the safety and efficacy of secondary non-penetrating deep sclerectomy (DS) after failed XEN gel stent implantation.

## 2. Materials and Methods

### 2.1. Study Design

This was an investigator-initiated, prospective, interventional study, conducted at a single tertiary glaucoma center. The study complies with the tenets of the Declaration of Helsinki and was approved by the local ethical committee (Institutional Review Board). Written informed consent was obtained from all included patients. The study was registered in the National Library of Medicine database (ClinicalTrials.gov identifier, NCT04381611).

### 2.2. Study Population

Consecutive eyes that underwent secondary DS with MMC following failed XEN gel stent implantation at the same institution (Glaucoma Research Centre, Montchoisi Clinic, Swiss Visio, Lausanne, Switzerland) between October 2015 and April 2018 were prospectively enrolled. Every effort was made to enroll all suitable patients as per the inclusion and exclusion criteria. Inclusion criteria were as follows: a diagnosis of primary or secondary open-angle glaucoma, previous XEN gel stent implantation carried out at the investigation center, uncontrolled glaucoma despite needling revisions and medical therapy. Glaucoma was defined as the association of repeatable visual field defects (persistent scotoma on at least two consecutive standard automated perimetry tests (Octopus, Haag Streit, Koeniz, Switzerland) with a test reliability index ≥15%) and an abnormal optic disc appearance (presence of neuroretinal rim thinning or localized or diffuse retinal nerve fiber layer defects) indicative of glaucoma, as observed under slit-lamp examination or on spectral-domain optical coherence tomography (SD-OCT) imaging (Spectralis OCT, Heidelberg Engineering AG, Heidelberg, Germany). Systematic gonioscopic examination was carried out to confirm angle opening. Glaucoma was considered as uncontrolled when functional and/or structural tests identified persistent signs of progression, or when IOP was persistently above the eye-specific target set by the treating ophthalmologist. The choice of the secondary procedure was left at the discretion of the treating surgeon. In addition, eyes with a post-operative follow-up shorter than 12 months were excluded from this analysis.

### 2.3. Primary Procedure: XEN Gel Stent

The XEN gel stent has a length of 6 mm, a 150-μm external diameter, and an inner lumen of 45 μm that was calculated using Hagen-Poiseuille law in order to avoid post-operative hypotony and achieve a resistance of 6–8 mmHg under physiological conditions of an aqueous production rate of 2 to 2.5 μL/min [13,14], The aim of the device is to create an artificial pathway through the trabecular meshwork and the sclera, allowing aqueous flow from the anterior chamber (AC) to the subconjunctival space.

All surgeries were conducted at the investigation center by one of two experienced surgeons (A.M. and K.M.), as either standalone or combined procedures, using a standardized ab interno technique previously detailed [7]. In all cases, intraoperative 0.1 mL MMC at a concentration of 0.02% was injected under Tenon’s capsule and spread with a microsponge applied to conjunctiva before the implant was injected. The MMC was not washed out. 

During the postoperative follow-up, if the treatment target IOP was not achieved after the first post-operative month, or if disease progression was noted, interventional treatment was performed as follows: If obstruction of the AC-tip of XEN Gel Stents was suspected, it was relieved by YAG fibrinolysis [15]; flat blebs were treated by needling revision procedures up to three times; and in other cases, IOP-lowering medications were re-introduced. In cases that were refractory to those measures, non-penetrating DS or glaucoma drainage device surgery was considered on an individual basis.

### 2.4. Secondary Procedure: Deep Sclerectomy

Enrolled patients all underwent secondary DS following failed primary XEN gel stent implantation.

Since the 1990s, DS has been recognized as a safer alternative to trabeculectomy, offering comparable success rates and minimizing the risk of postoperative complications [16,17,18,19,20]. The essential difference with trabeculectomy is the non-penetrating nature of DS, through the creation of a filtration membrane, the trabeculo-Descemet’s membrane (TDM). Moreover, in DS, the excision of the inner scleral flap creates an intrascleral lake, potentially increasing aqueous flow through intrascleral and suprachoroidal pathways, in addition to subconjunctival filtration [21,22,23,24].

All the surgical procedures were performed by one of the same glaucoma surgeons who initially performed XEN gel stent implantation (A.M. or K.M.) [25]. When a different site could be selected, the XEN gel stent was left in place. Otherwise, when it was not possible to avoid the old surgical site, the device was removed following conjunctival opening. Three surgical sponges soaked with 0.2 mg/mL MMC were inserted under the conjunctiva for 2–3 min before the scleral dissection. After the sponges were removed, washout was performed. No case had to be converted to a trabeculectomy because of perforation of the TDM.

Beyond the first post-operative month, when the filtration through the TDM was considered to be insufficient because of elevated IOP, a laser goniopuncture (LGPT) was performed with the neodymium (Nd):YAG laser in the anterior thinnest portion of the TDM. After the LGPT opening of the TDM, if the treatment target IOP was not achieved, needling revisions were performed. After the needling revision, IOP-lowering medications were reintroduced postoperatively if the patient’s target IOP was not reached.

### 2.5. Outcome Measures

Success of the secondary procedure was defined either as complete, if the unmedicated IOP at last follow-up visit was ≤12 mmHg, ≤15 mmHg, or ≤18 mmHg, both with and without a relative IOP reduction ≥20% or more, compared to the last IOP prior to reoperation (DS), or as qualified if the IOP met the same thresholds with fewer medications than immediately before DS. Loss of light perception, serious irreversible complications, IOP over 18 mmHg, or any subsequent glaucoma surgical intervention following DS were considered surgical failures. Further drainage or filtering surgery, surgical revisions, and cyclodestruction were all considered reoperations, and as such, failure of the procedure. LGPTs and needling procedures were not considered reoperations. Secondary efficacy and safety outcome measures included the mean reduction in IOP and topical hypotensive medications, the number of LGPTs and needling revisions required to maintain IOP within individual target ranges, and the rate of surgical failure. Safety endpoints included the rate of intraoperative complications and post-operative AEs during the entire follow-up.

### 2.6. Statistical Analysis

Descriptive statistics included mean and standard deviation (SD) for normally distributed variables, and median and interquartile range (IQR) for non-normally distributed variables. Kaplan–Meier survival curves were used to assess the cumulative probability of success and failure. Baseline IOP was defined as the mean of the last two preoperative measures. Associations between failure and demographic or clinical variables such as age, gender, ethnicity, diagnosis, or number of preoperative treatments or surgeries were assessed. All tests were two-tailed and a *p*-value less than 0.05 was considered statistically significant. Statistical analyses were performed with commercially available software (StataCorp, College Station, TX, USA).

## 3. Results

### 3.1. Baseline Characteristics of Study Population

Out of a total of 149 eyes that underwent XEN standalone or XEN plus phacoemulsification surgery between January 2015 and June 2016, 24 eyes needed subsequent glaucoma interventions because of clinical evidence of failing bleb with elevated IOP, above the individual target range. Out of those, 17 (70.8%) were deemed suitable to undergo secondary MMC-augmented DS and were enrolled in this study. Among the others, 3 eyes (12.5%) underwent surgical bleb revision, 2 (8.3%) had a second XEN gel stent implantation, 1 (4.2%) underwent placement of a Baerveldt glaucoma drainage device (Abbot Inc., Lake Bluff, IL, USA) augmented with the XEN gel stent, and 1 (4.2%) underwent surgical reposition of the XEN gel stent. Two patients were lost to follow-up before their 12-month appointment. Sufficient clinical data were thus available from 15 eyes (62.5%) of 14 patients. These patients were considered eligible for analysis. As only one subject (7.14% of the entire cohort population) had both eyes eligible for the analysis, a statistical correction was not applied for the presence of two eyes from the same patient.

The mean ± SD follow-up was 20.1 ± 4.9 months (range 12 to 24). The mean age of the study population at enrolment was 72.1 ± 8.2 years, 66.7% (*n* = 10) were female, and 80% (*n* = 12) were Caucasians. In all, 33.3% had a diagnosis of POAG, followed by PEXG (26.6%). The primary XEN gel stent implantation had been a standalone procedure for 5 eyes (33.3%); the remainder were combined with phacoemulsification (10 eyes, 66.7%). The average time of failure for primary XEN gel stent was 11.1 ± 7.6 months (range, 1 to 28 months). Glaucoma severity as per Hodapp–Parrish–Anderson criteria ranged from mild-to-moderate, with a mean visual field MD of 5.3 ± 2.9 dB at enrolment. Baseline characteristics of the study patients are summarized in Table 1.

### 3.2. Safety 

No intraoperative complications were noted among the studied cohort, neither at time of primary XEN gel stent surgery nor at time of secondary DS. None of the combined procedures were associated with posterior capsule rupture or the need for anterior vitrectomy.

### 3.3. Intraocular Pressure, Medication Use and Visual Acuity 

**Figure 1:**
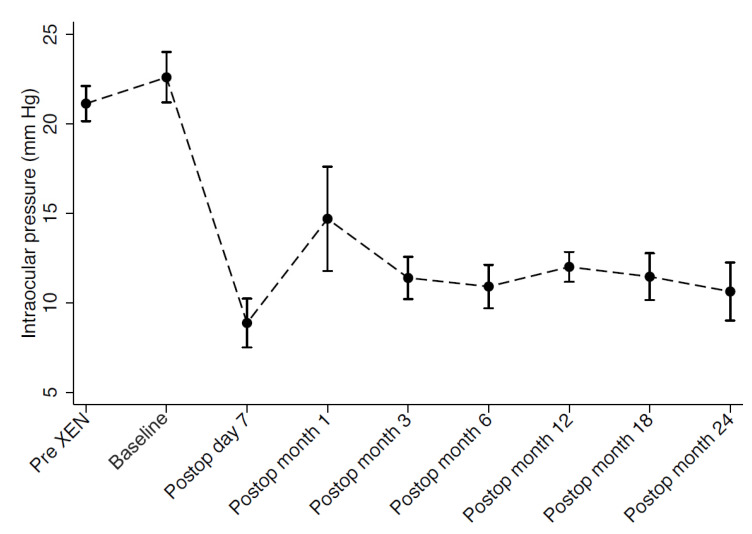
Graph showing mean intraocular pressure through 24 months of follow-up.

**Figure 2:**
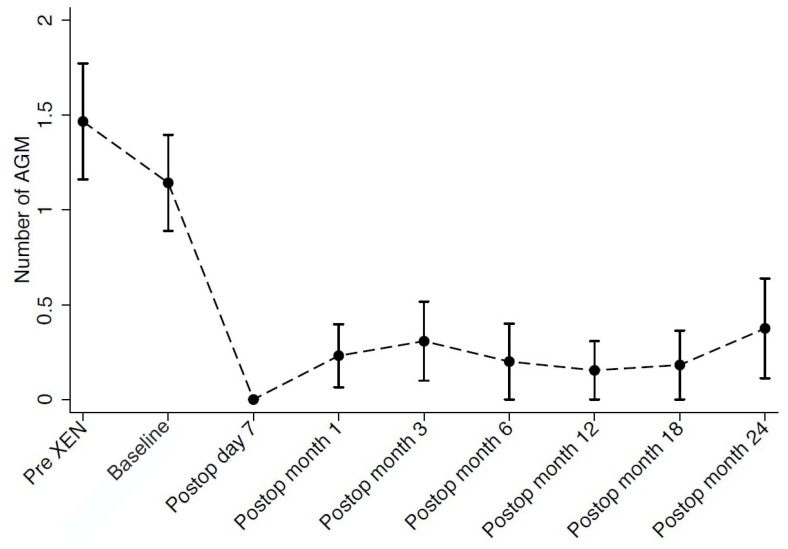
Graph showing the number of antiglaucoma medications (AGM) through 24 months of follow-up.

Mean medicated IOP before XEN gel stent surgery (pre-XEN), before DS (baseline), and at last follow-up visit after DS were 21.1 ± 3.7 mmHg, 22.6 ± 5.3 mmHg, and 12.3 ± 5.5 mmHg, respectively. Overall, we observed a reduction of 45.6% in IOP between the time of XEN failure and the last follow-up visit after DS (*p* < 0.001). The number of anti-glaucoma medications concomitantly dropped from 1.5 ± 1.1 (pre-XEN) and 1.1 ± 0.9 (baseline) to 0.3 ± 0.7, representing a reduction of 72.7% following secondary DS (*p* = 0.014). At the last follow-up visit, 13.3% of eyes (*n* = 2) required antiglaucoma medications to achieve target IOP. Antiglaucoma medications and IOP progression throughout the follow-up period are presented in Figure 1 and Figure 2. Mean BCVA at last follow-up visit after DS remained statistically unchanged compared to BCVA at baseline (0.9 ± 0.2 decimals).

### 3.4. Primary Outcome: Surgical Success

**Figure 3:**
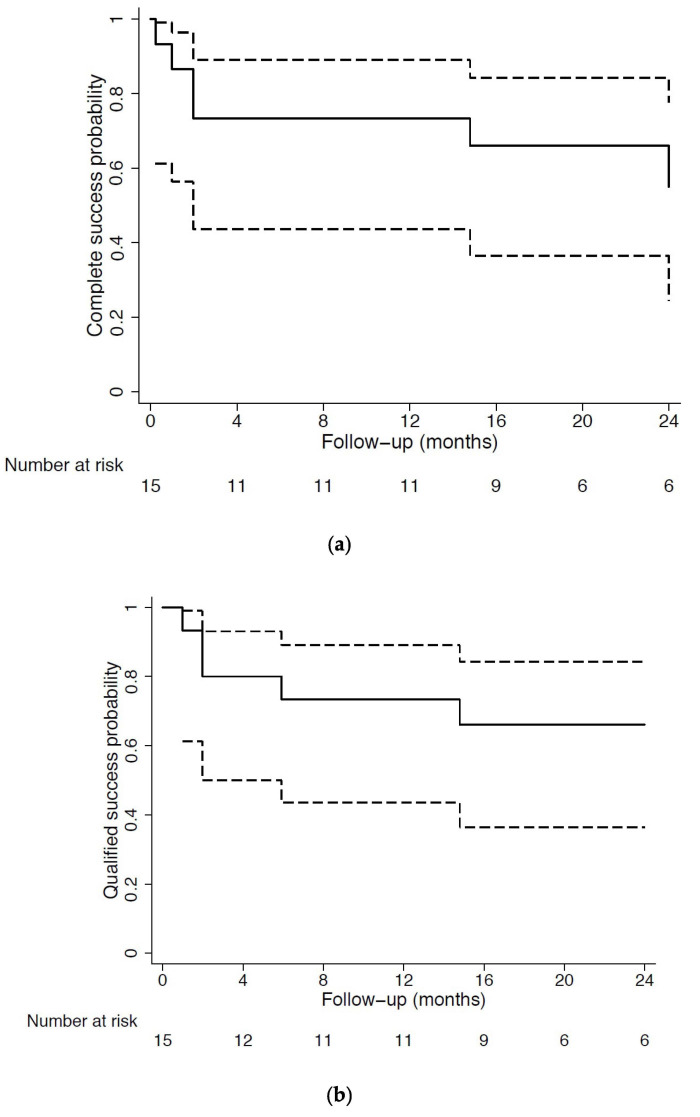
Cumulative probability of complete (**a**) and qualified (**b**) success (Kaplan–Meier curves) using the 15 mmHg or less intraocular pressure threshold.

Complete success at last follow-up visit was achieved in 40% of eyes using the strictest threshold of 12 mmHg or less with a concomitant 20% IOP reduction from baseline, whereas 60% of eyes achieved an unmedicated IOP of 15 mmHg or less and 66.7% achieved an unmedicated IOP of 18 mmHg or less. Qualified success was obtained in 46.7% of eyes using the 12-mmHg or less and 20% IOP reduction from baseline definition, while 66.7% and 80% of eyes reached a medicated IOP of 15 mmHg or less and 18 mmHg or less, respectively. The Kaplan–Meier survival curves are presented in Figure 3. Out of 15 eyes, 3 (20%) were classified as complete failure due to an uncontrolled intraocular hypertension above 18 mmHg, despite medical treatment and needling revisions, which required further surgical intervention. Among reoperated eyes, 2 underwent surgical bleb revision, and one underwent implantation of the eyeWatch system (Rheon Medical, Lausanne, Switzerland) [26]. The average time of complete failure was 6.3 ± 6.1 months after secondary surgery. Table 2 presents the surgical success and failure rates against all definitions. Association analysis showed no statistically significant association between surgical outcome and any of the patients’ demographics or recorded clinical data.

### 3.5. Postoperative Interventions

After DS, needling revisions were performed in 46.7% (*n* = 7) of eyes; 85.7% of them (*n* = 6) required a single intervention to control their IOP, while 14.3% (*n* = 1) required two needling interventions over the entire follow-up period. On average, the first needling intervention was performed 6.6 ± 7.1 months after surgery. The only patient who required more than one needling treatment underwent the procedure at 6 and 24 months, postoperatively. Laser goniopuncture was performed in nine eyes (60.0%), including all eyes that subsequently required a needling intervention. Postoperative interventions are summarized ion Table 3.

### 3.6. Postoperative Complications

Three eyes (20.0%) experienced refractory intraocular hypertension, requiring further surgery, two eyes (13.3%) experienced persistent bleb leakage and required conjunctival sutures. The latter occurred at a mean post-operative time of 1.5 ± 0.5 months. One eye (6.7%) experienced persistent hypotony, defined as IOP persistently <5 mmHg without evidence of bleb leakage, choroidal detachment, folds, or loss of visual acuity. This case of hypotony resolved following a 1-month topical treatment of dexamethasone and bromhydrate scopolamine, three times a day. Postoperative complications are reported in Table 4.

## 4. Discussion

The current study represents, to the best of our knowledge, the first prospective study describing the outcomes of DS with MMC following failed XEN gel stent with MMC surgery, with a long-term postoperative follow-up. Its results suggest that failed primary XEN implantation may not affect the safety or efficacy outcomes of secondary filtering surgery. Although, in our experience, performing secondary DS was marginally more challenging than a primary procedure, there was a relatively low rate of postoperative AEs following the reoperation. Moreover, no serious sight-treating complications were observed. In terms of efficacy, a good long-term IOP-lowering effect was achieved, with a mean IOP reduction of 45.6% from the medicated IOP levels at the timing of XEN failure. Meanwhile, a concomitant and significant decrease (−72.7%) in antiglaucoma medications was observed. The number of antiglaucoma medications prior to DS seems to be very low (1.1 ± 0.9). The reason behind that probably reflects the Swiss Medical Care System and the real-life environment of the present study. Indeed, the time the patient had to wait from the moment a diagnosis of a failed XEN had been made and the subsequent reoperation is very low (sometimes only days or weeks), so in most of the cases it was not even necessary to give extra antiglaucoma medications to the patient. Furthermore, the rates of surgical failure due to uncontrolled IOP requiring subsequent glaucoma procedures within 24 months (20%) was within the reported rates for primary DS [27,28,29].

In recent years, studies have demonstrated that, although the success rates of XEN gel stents remain reasonably high at 24 months, its success rates gradually decrease over time. Mansouri et al. [6,7] observed a complete success rate of 62.4% at 1 year versus 51.9% at 2 years, using an 18 mmHg IOP threshold. Our group has identified increasing rates of reoperation (6% at 1 year vs. 11.4% at 2 years) rates that were also observed by other research groups [4,6,7]. These rates were shown to be even higher in Black and Afro-Latino populations, with up to 40% requiring secondary glaucoma surgery by 12 months [30]. Moreover, several studies have found that stent-related complications such as blockage of the internal lumen [15,31], device degradation [32], or device movements [33] can occur months to years after implantation, with a subsequent need of surgical reintervention.

Despite ample data on the frequency and causes of failure of the XEN gel stent, there is a paucity of evidence on how to manage glaucoma patients once this technique fails. Gizzi et al. [11] demonstrated that MMC-augmented trabeculectomy following failed XEN gel stent surgery is technically feasible and effective in lowering IOP. Nevertheless, they observed a significant incidence of early-onset bleb leaks (37.5%), a high rate of hypotony (25%) leading to frequent shallow choroidal detachment (12.5%), and chorioretinal macular folds (12.5%) with resulting visual loss (50% losing two Snellen lines). In comparison, the present study suggests that secondary DS is safer than secondary trabeculectomy following failed XEN gel stent implantation. These conclusions are in keeping with the results of studies and meta-analyses comparing primary DS and primary trabeculectomy [34,35]. Moreover, the results of the present study are similar in terms of IOP reduction, medication reduction, and complication rate to the reported outcomes of primary DS, supporting the assumption that XEN gel stent implantation as a primary procedure has little to no effect on the outcome of subsequent DS [25,36,37,38,39].

However, analyses of secondary procedures are required due to the prolonged and cumulative tissue exposure to MMC during the XEN implantation and the subsequent procedures, which might increase the long-term rates of bleb-related complications and altered ciliary body function. For these reasons, it may be advisable that secondary procedures use lower MMC concentrations or exposure time, or less potent antimetabolites such as 5-fluorouracil. Nevertheless, the present study reports low rates of bleb complications despite the use of intraoperative MMC in similar doses to those generally used in trabeculectomy. As a result, we hypothesize that the difference in safety lies in the nature of the two filtering techniques used. Indeed, it has been widely shown that DS is associated with a lower rate of postoperative complications compared to trabeculectomy [16,17,18,19,20], and may have some advantages in high-risk-of-failure eyes. The main technical differences are the non-penetration of the anterior chamber intraoperatively, and the removal of the deeper scleral flap during the DS. The creation of a filtration membrane, the TDM, is responsible for the gradual reduction of the IOP, intra- and postoperatively. Excessive flow in the early postoperative period was suspected to contribute to a number of complications of trabeculectomy. The preservation of the TDM in DS acts as a protective mechanism with this regard. In addition, it was shown that the non-penetrative nature of DS reduces the amount of intraocular inflammation, which is known to contribute to the failure of filtering surgery and may compromise bleb survival [40,41,42]. After primary DS, shallow AC, hypotony maculopathy, and AC inflammation are infrequent. Furthermore, the excision of the deeper scleral flap creates an additional outflow pathway by forming an intrascleral lake, which is believed to potentiate suprachoroidal flow and reduce pressure on the subconjunctival bleb [21,22,23,24]. These mechanisms are thought to further contribute to creating a more diffuse and posterior bleb morphology compared to that achieved through trabeculectomy. All those features are probably responsible for the lower rate of bleb leakage found in the present study (13.3% vs 37.5% after secondary trabeculectomy) [11].

Previously, Laroche et al. [12] used a Baerveldt tube in order to rescue a failed XEN gel stent via a technique previously described for refractory glaucoma [43]. In a patient with a failed XEN gel stent, a 250-Baerveldt tube was inserted in the superonasal quadrant and positioned to be connected with the present XEN implant. The double lumen was then sutured to secure the position. The follow-up of this case report reached only one month postoperatively, reporting an unmedicated IOP of 5 mmHg. Although the XEN-augmented Baerveldt technique constitutes a new and innovative variation of a standard glaucoma drainage device (GDD), by lowering the risks of complications traditionally associated with tube surgeries, such as early postoperative hypotony or long term corneal endothelial cell loss, its relevance to rescuing failed XEN devices appears less relevant. Indeed, patients selected for XEN gel stent implantation generally suffer from early-to-moderate glaucoma and are unlikely to require a device usually reserved for more advanced or refractory cases for a number of years, which may be beyond the XEN gel stent’s lifespan. Second, GDD surgery is characterized by a high technical difficulty. In addition, prospective studies on XEN-augmented Baerveldt implantation have reported a high rate of failure and reoperation at 12 months, even if in non-refractory eyes [44,45]. On the other hand, XEN-augmented Baerveldt technique could prove useful to rescue a failed XEN gel stent in cases where conjunctiva is not deemed adequate for filtering surgery.

### Study Limitations

The present study has several limitations. First, it was not a randomized controlled comparative study and there was no control group. In particular, the absence of a control group with other filtering or cyclophotoablative surgical procedures seems to be a strong limitation for the value of the results and can be partially explained by the design of the study and by the treatment algorithm used by the surgeons, who generally apply a non-penetrating glaucoma surgery after a failed MIGS. Another limitation was the absence of a preoperative medication washout. This can be explained by the uncontrollable nature of glaucoma in the enrolled cohort, associated with the risk of disease progression over the washout period. Furthermore, the nature of the studied indication implies that only a relatively small number of patients met the inclusion criteria, leading to a potential size bias. The fact that all cases were operated by the same two surgeons, in a single tertiary glaucoma center, may be considered both a limitation and a strength. Finally, another limitation of our study is that it was conducted in a predominantly homogenous (Caucasian) population. More research is needed to evaluate the success of secondary procedures in the longer term and in other ethnicities.

## 5. Conclusions

The present study suggested that failed MMC-augmented XEN gel stent implantation does not affect the outcomes of subsequent filtering surgery. Furthermore, it demonstrated that secondary deep sclerectomy with MMC following failed MMC-augmented XEN gel stent implantation produced a significant and sustained IOP reduction over 24 months postoperatively. Moreover, this surgical approach remains relatively safe in high-risk-of-failure eyes and seems to display higher success rates and lower rates of AEs compared to MMC-augmented trabeculectomy and XEN-augmented Baerveldt techniques used for the same indication.

## Figures and Tables

**Table 1:** Baseline demographics and clinical characteristics of study population.

Demographic and Clinical Data	Mean ± SD (%)
Age (years)	72.1 ± 8.2
Range	53–89
Female gender	10 (66.7%)
Study eye	
Right	8 (53.3%)
Left	7 (46.6%)
Ethnicity	
Caucasian	12 (80%)
Black	2 (13.3%)
Asian	1 (6.7%)
Bilateral cases	1
Diagnosis	
POAG	5 (33.3%)
PEXG	4 (26.6%)
Pigmentary glaucoma	3 (20%)
Other	3 (20%)
Central corneal thickness (μm)	540.1 ± 53.7
pre-XEN Visual field (dBs)	
MD	5.3 ± 2.9
sLV	3.9 ± 1.9
pre-XEN OCT RNFL thickness (μm)	85.6 ± 16.5
pre-XEN BCVA (decimal)	0.8 ± 0.3
Baseline BCVA (decimal)	0.9 ± 0.2
pre-XEN IOP (mmHg)	21.1 ± 3.7
Baseline IOP (mmHg)	22.6 ± 5.3
pre-XEN Medications	1.5 ± 1.1
Baseline Medications	1.1 ± 0.9

BCVA = best-corrected visual acuity; dB = decibels; IOP = intraocular pressure; MD = mean deviation; POAG = primary open-angle glaucoma; PEXG = pseudoexfoliation glaucoma; RNFL = retinal nerve fiber layer; SD = standard deviation; sLV = square of loss of variance.

**Table 2:** Surgical success and failure rates against all definitions.

Definition	Percentage
Complete success (unmedicated)	
Intraocular pressure ≤ 12 mmHg	40
With a reduction of more than 20% from baseline	40
Intraocular pressure ≤ 15 mmHg	60
With a reduction of more than 20% from baseline	60
Intraocular pressure ≤ 18 mmHg	66.7
With a reduction of more than 20% from baseline	66.7
Qualified success (medicated)	
Intraocular pressure ≤ 12 mmHg	46.7
With a reduction of more than 20% from baseline	46.7
Intraocular pressure ≤ 15 mmHg	66.7
With a reduction of more than 20% from baseline	66.7
Intraocular pressure ≤ 18 mmHg	80
With a reduction of more than 20% from baseline	80
Complete failure	20

**Table 3:** Postoperative interventions during the follow-up.

Postoperative Interventions	Percentage
Total of needling revisions	46.7
# 1 needling revision	85.7
# 2 needling revisions	14.3
Laser Goniopuncture	60

**Table 4:** Postoperative complications during the follow-up.

Postoperative Complications	Percentage
Refractory intraocular hypertension requiring further surgery	20
Persistent bleb leakage requiring conjunctival sutures	13.3
Persistent hypotony, defined as IOP persistently <5 mmHg	6.7

## Data Availability

The datasets used and/or analyzed during the current study are available from the corresponding author on reasonable request.

## Abbreviations

DS	deep sclerectomy
MMC	mitomycin-C
IOP	intraocular pressure
MIGS	minimally invasive glaucoma surgery
POAG	primary open angle glaucoma
PEXG	pseudoexfoliative glaucoma
SD-OCT	spectral-domain optical coherence tomography
AC	anterior chamber
TDM	trabeculo-Descemet’s membrane
LGPT	laser goniopuncture
SD	standard deviation
IRQ	interquartile range
MD	mean deviation
sLV	square of loss of variance
RNFL	retinal nerve fiber layer
BCVA	best corrected visual acuity
AGM	antiglaucoma medications
dB	decibel
AE	adverse event
GDD	glaucoma drainage device
